# Bladder Tissue Microbiome Composition in Patients of Bladder Cancer or Benign Prostatic Hyperplasia and Related Human Beta Defensin Levels

**DOI:** 10.3390/biomedicines10071758

**Published:** 2022-07-21

**Authors:** Bassel Mansour, Ádám Monyók, Márió Gajdács, Balázs Stercz, Nóra Makra, Kinga Pénzes, István Vadnay, Dóra Szabó, Eszter Ostorházi

**Affiliations:** 1 Department of Urology, Markhot Ferenc University Teaching Hospital, 3300 Eger, Hungary; bassel.mansour0@gmail.com (B.M.); adammonyok@gmail.com (Á.M.); 2 Department of Oral Biology and Experimental Dental Research, Faculty of Dentistry, University of Szeged, 6720 Szeged, Hungary; mariopharma92@gmail.com; 3 Department of Medical Microbiology, Semmelweis University, 1089 Budapest, Hungary; stercz.balazs@med.semmelweis-univ.hu (B.S.); makra.nora@med.semmelweis-univ.hu (N.M.); penzes.kinga@med.semmelweis-univ.hu (K.P.); szabo.dora@med.semmelweis-univ.hu (D.S.); 4 Department of Pathology, Markhot Ferenc University Teaching Hospital, 3300 Eger, Hungary; vadnay.istvan@gmail.com; 5 Department of Dermatology, Venereology and Dermatooncology, Semmelweis University, 1089 Budapest, Hungary

**Keywords:** bladder cancer, bladder urothelium, prostatic hyperplasia, microbiome, human beta defensins, urine

## Abstract

Balance between the microbiome associated with bladder mucosa and human beta defensin (HBD) levels in urine is a dynamic, sensitive and host-specific relationship. HBD1—possessing both antitumor and antibacterial activity—is produced constitutively, while the inducible production of antibacterial HBD2 and HBD3 is affected by bacteria. Elevated levels of HBD2 were shown to cause treatment failure in anticancer immunotherapy. Our aim was to assess the relationship between microbiome composition characteristic of tumor tissue, defensin expression and HBD levels measured in urine. Tissue samples for analyses were removed during transurethral resection from 55 bladder carcinoma and 12 prostatic hyperplasia patients. Microbiome analyses were carried out with 16S rRNS sequencing. Levels of HBD mRNA expression were measured with qPCR from the same samples, and urinary amounts of HBD1, 2 and 3 were detected with ELISA in these patients, in addition to 34 healthy volunteers. Mann–Whitney U test, Wilcoxon rank sum test (alpha diversity) and PERMANOVA analysis (beta diversity) were performed. Defensin-levels expressed in the tumor did not clearly determine the amount of defensin measurable in the urine. The antibacterial and antitumor defensin (HBD1) showed decreased levels in cancer patients, while others (HBD2 and 3) were considerably increased. Abundance of *Staphylococcus*, *Corynebacterium* and *Oxyphotobacteria* genera was significantly higher, the abundance of *Faecalibacterium* and *Bacteroides* genera were significantly lower in tumor samples compared to non-tumor samples. *Bacteroides*, *Parabacteroides* and *Faecalibacterium* abundance gradually decreased with the combined increase in HBD2 and HBD3. Higher *Corynebacterium* and *Staphylococcus* abundances were measured together with higher HBD2 and HBD3 urinary levels. Among other factors, defensins and microorganisms also affect the development, progression and treatment options for bladder cancer. To enhance the success of immunotherapies and to develop adjuvant antitumor therapies, it is important to gain insight into the interactions between defensins and the tumor-associated microbiome.

## 1. Introduction

Bladder cancer (BC) is the fourth most common cancer in men and 11th in women with a high prevalence and global incidence. Approximately 3.0% of all new cancer diagnoses and 2.1% of all cancer deaths are due to urinary bladder cancer [1]. Based on stage classification and risk assessment—in addition to surgical removal—therapeutic procedures include Bacillus Calmette–Guérin (BCG) installation, radiation therapy, chemotherapy and the use of checkpoint inhibitors [2].

The most important risk factors for the development of bladder carcinoma (BC) are smoking, exposure to aromatic amines and many other toxic compounds of environmental origin, such as arsenic in drinking water or insecticides [3,4,5]. These carcinogenic toxins are excreted from the bloodstream through the kidneys and they interact with the microbiota during ensuing storage in the bladder. The resulting metabolites may increase or decrease the risk of BC. Bacteria present in the bladder are one of the many contributors leading to the development and progression of BC [6], with several studies describing microbiome changes associated with BC [7,8,9,10].

The physiological balance between the microbiome and mucosal defensin-levels is dynamic, sensitive and host-specific. Members of the microbiome affect the levels of inducible human β-defensin 2 (HBD2) and HBD3, however, the production of HBD1 is constitutive and host-dependent. Mucosal defensin-levels select for bacteria that make up the microbiome, based on strain-specific sensitivity. Defensins, as antibacterial peptides, achieve their bactericidal effect primarily by breaking down the integrity of the outer membrane of Gram-negative bacteria. A healthy individual is characterized by a genetically determined level of HBD1, while the feedback mechanism of the colonizing microbes and the amounts of HBD2–3 that control each other creates the steady state between the microorganisms and antibacterial peptides. The autonomous defensin production of tumor cells may also amend this state to an unpredictable degree and direction.

Several studies have demonstrated the antitumor efficacy of HBD1 against malignant processes in various organs [11,12,13], as well as its protective effect against BC [14,15]. Chang et al. noted the detrimental effect of increased levels of HBD2 on BCG therapy against BC, and later confirmed the exact mechanism of action through Toll-like receptors [16,17]. It is well-known that not only the change in the gut microbiome affects the effectiveness of the anti-tumor checkpoint inhibitors, such as pembrolizumab or nivolumab, but the bacteria present in the urogenital system can also directly inhibit this therapy [18]. When planning individual therapies, it is essential to have more in-depth knowledge about the components of the bladder tumor microbiome and the HBD levels associated with them.

We previously demonstrated that the microbiome composition of bladder tumor tissue samples is significantly different from the planktonic microbiome content of the urine surrounding the tumor [19]. *Akkermansia*, *Bacteroides*, *Clostridium sensu stricto*, *Enterobacter* and *Klebsiella*, as “five suspect genera”, were over-represented in tumor tissue samples compared to the microbiota composition of the urine. However, taxa with a high abundance in tumor tissue are not necessarily tumor-associated bacteria; they may just occur more often adhering to the mucosal layer. In our present study, we sought to determine whether there is a difference in the microbiome composition of tumor and non-tumor bladder tissue samples, the amounts of HBD1, HBD2 and HBD3 mRNA expressed in them and the levels of defensins found in the urine of cancer and non-cancer patients. We compared the tissue microbiome of BC patients (BC group) with the microbiome of mucosal samples from patients with benign prostatic hyperplasia (PH group), the levels of HBD mRNA expressed in tissue pieces and the levels of defensins measured in the urine of BC and PH patients, compared to healthy volunteers (HV group).

## 2. Materials and Methods

### 2.1. Sample Collection

The present microbiome-defensin molecular study included 55 patients with BC and 12 patients with PH. Mucosal tissue samples removed during transurethral resection (TUR) were divided for histological and microbiome-defensin molecular analysis. Urine samples were collected directly from the bladder during TUR surgery for quantitative defensin detection and traditional routine culture. Spontaneously excreted urine was examined from 34 healthy volunteers. Exclusion criteria were urinary infection, antibiotic or probiotic treatment two months prior to surgery. The characteristics of the study participants are presented in Supplementary Table S1. Members of the PH group were chosen in such a way that they matched the members of the BC group in most characteristics (with the exception of gender).

### 2.2. DNA Isolation, 16S rRNA Gene Library Preparation and MiSeq Sequencing

From tissue samples DNA isolation was performed by ZymoBIOMICS DNA Miniprep Kit (Zymo Research Corp., Irvine, CA, USA), after enzymatic dissolution with ProtK (56 °C, 5 h). Bacterial DNA was amplified with tagged primers covering the V3–V4 region of bacterial 16S rRNA gene. PCR and DNA purifications were performed according to Illumina’s protocol. PCR product libraries were assessed using DNA 1000 Kit with Agilent 2100 Bioanalyzer (Agilent Technologies, Waldbronn, Germany). Equimolar concentrations of libraries were pooled and sequenced on an Illumina MiSeq platform (Illumina, San Diego, CA, USA) using MiSeq Reagent Kit v3 (600 cycles PE).

To evaluate the contribution of extraneous DNA from reagents, extraction negative controls and PCR negative controls were included in every run. To ensure reproducibility, all analysis procedures were done in triplicate from 3 separately isolated DNA samples from each patient. Raw sequencing data were retrieved from Illumina BaseSpace and data were analyzed using the CosmosID [20] bioinformatics platform. 

### 2.3. Defensin Expression Assays

Total RNA from tissue samples was isolated by innuPREP RNA Mini Kit 2.0 (Analytik Jena GmbH, Jena, Germany) according to the manufacturer’s instructions after enzymatic dissolution with ProtK (56 °C, 5 h). Furthermore, 80–100 ng of RNA was used for RT-PCR assay performed using the PrimeScript RT reagent kit (Takara Bio, San Jose, CA, USA) and the resulting cDNA was amplified on a qTOWER 3G (Analytik Jena GmbH, Jena, Germany) instrument in the presence of selected primers. The following primers were used for defensin expression assays: HBD1: 5′-TTG TCT GAG ATG GCC TCA GGT AAC-3′ forward, 5′-ATA CTT CAA AAG CAA TTT TCC TTT AT-3′ reverse, HBD2: 5′-CCAGCCATCAGCCATGAGGGTCTTG-3′ forward, 5′-CAT GTC GCA AGT CTC TGA TGA GGG AGG-3′ reverse, HBD3: 5′-AGC CTA GCA GCT ATG AGG ATC-3′ forward and 5′-CTT CGG CAG CAT TTT CGG CCA-3′ reverse. The primers for the GAPDH housekeeping gene were 5′-CTA CTG GCG CTG GCA AGG CTG T-3′ forward and 5′-GCC ATG AGG TCC ACC ACC CTG CTG-3′ reverse.

Relative changes in mRNA expression were calculated by using the double delta Ct (ΔΔCt) method [21]. ΔCt of each tumor and PH sample was normalized, calculating the difference between their gene of interest Ct value (HBD1, HBD2 or HBD3) and their Ct value for the GAPDH housekeeping gene. ΔΔCt values were calculated using the median ΔCt value of PH samples considered as controls. The relative expression (RQ) fold change was calculated as 2^−ΔΔCt^.

### 2.4. ELISA (Enzyme Linked Immunosorbent Assay)

For quantitative measurement of human beta defensins in urine, the following ELISA kits were used, according to manufacturer instructions: SEB373Hu for HBD1, SEA072Hu for HBD2 and SEE132Hu for HBD3 (Cloud-Clone Corp., Houston, TX, USA). All diluted standards, samples and blank wells were measured in duplicate.

### 2.5. Statistical Analysis

Levels of statistical significance (*p* < 0.05) for the difference between urine defensin levels, defensin expression rate and bacterial taxa abundances measured in the different cohorts was calculated by Mann–Whitney U test. Statistical significance between cohorts were implemented by Wilcoxon rank sum test for Chao1 alpha diversity and PERMANOVA analysis for Jaccard principal coordinate analysis (PCoA) beta diversity using the statistical analysis support application, CosmosID [20].

### 2.6. Ethical Considerations

Sample collection protocols were approved by the Ethics Committee (EC) of Markhot Ferenc University Teaching Hospital (MFUTH) and EC of Semmelweis University (SE RKEB: 100/2018/100-1/2018/2021). The study was conducted in accordance with the Declaration of Helsinki ethical standards that promote and ensure respect and integrity for all human subjects. Patients treated at the MFUTH’s Urology Department between January and July of 2021 were enrolled in this study. The healthy volunteers (HV) were employees and students at Semmelweis University who volunteered for the urine test. All research was performed in accordance with guidelines and regulations of MFUTH. Written informed consent was obtained from all patients.

All research was performed in accordance with guidelines and regulations of MFUTH. Written informed consent was obtained from all patients to participate in the study. All study participants gave written informed consent that data from their personal test results could be published. All data and test results in the manuscript cannot be linked to the individual participants, all tests were anonymized.

## 3. Results

From 55 BC and 12 PH tissue samples, three smaller pieces were processed separately, for a total of *n* = 201 tested samples from 67 patients. Following 16S rRNA sequencing, a total of 52.8 million valid bacterial sequences were obtained, resulting in 40.7 million high-quality reads. The median number of reads in BC tissue samples was significantly higher (271,525 (IQR: 44,506)) (*p* = 0.001) than in PH bladder urothelial samples (110,083 (IQR: 16,711)).

**Figure 1:**
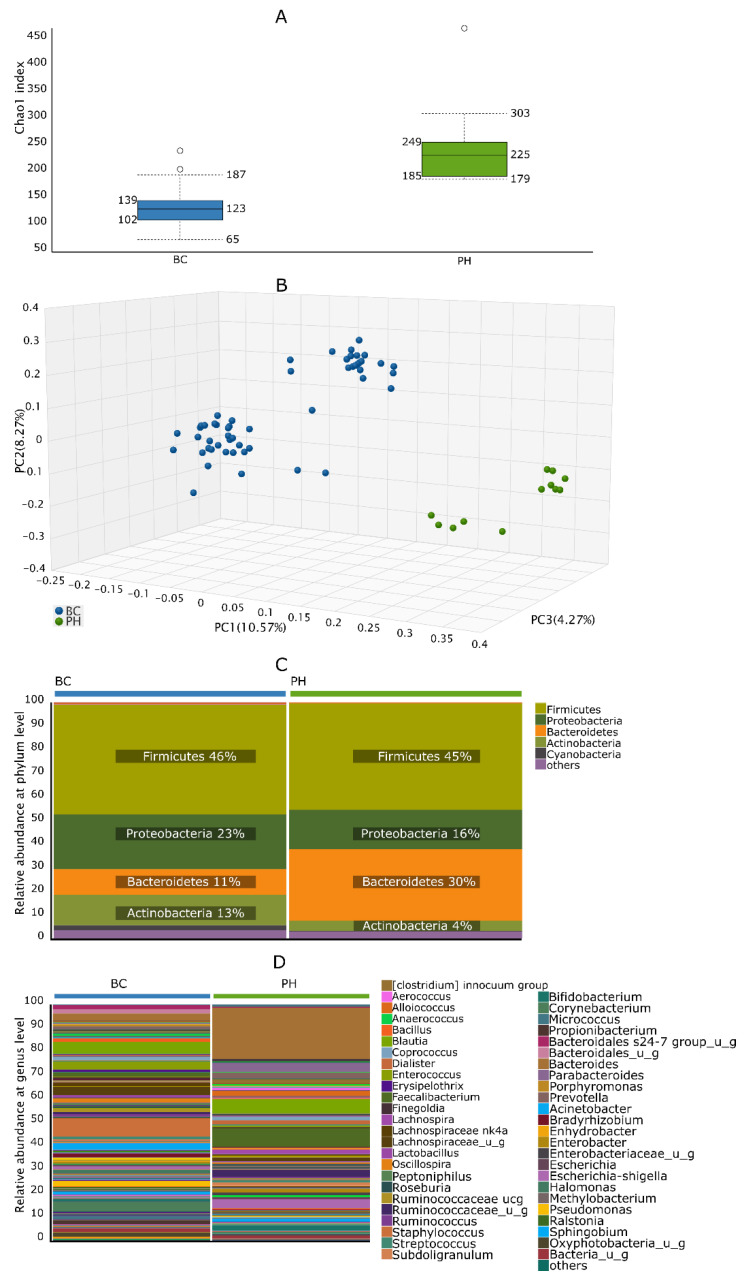
Comparison of tissue microbiome composition of bladder cancer (BC) and prostatic hyperplasia (PH) patients. (**A**) Chao1 alpha diversity at genus level, (**B**) Jaccard beta diversity at genus level, (**C**) taxa abundance in cohorts at phylum level, (**D**) taxa abundance in cohorts at genus level.

Although BC tissues contained higher amounts of bacterial DNA than PH urothelial samples, alpha diversity of the tumor-specific microbiome was still significantly (*p* < 0.001) lower than that of non-tumor samples (Figure 1A). Beta diversity PCoA also showed significant (*p* = 0.001) difference between the two cohorts (Figure 1B). At phylum level, the most abundant taxa in the BC and PH groups were Firmicutes (46% vs. 45%; *p* > 0.05), Proteobacteria (23% vs. 16%; *p* = 0.006), Actinobacteria (13% vs. 4%; *p* < 0.001) and Bacteroidetes (11% vs. 30%; *p* < 0.001) (Figure 1C). Median abundance of bacteria belonging to the Cyanobacteria phylum was significantly higher in the BC group (*p* = 0.011). The most striking differences between BC and PH groups were in the abundance of Staphylococcus (7.89% vs. 0.59%; *p* < 0.001), Corynebacterium (3.83% vs. 0.63%; *p* = 0.001), Faecalibacterium (1.92% vs. 7.79%; *p* < 0.001) and Bacteroides (3.22% vs. 21.54%; *p* < 0.001) (Figure 1D). Although Oxyphotobacteria (from the Cyanobacteria phylum) did not belong to the predominant bacterial genus in the microbiome, there was significant difference in their abundance between the BC and PH groups (2.11% vs. 0.07%; *p* = 0.024).

**Figure 2:**
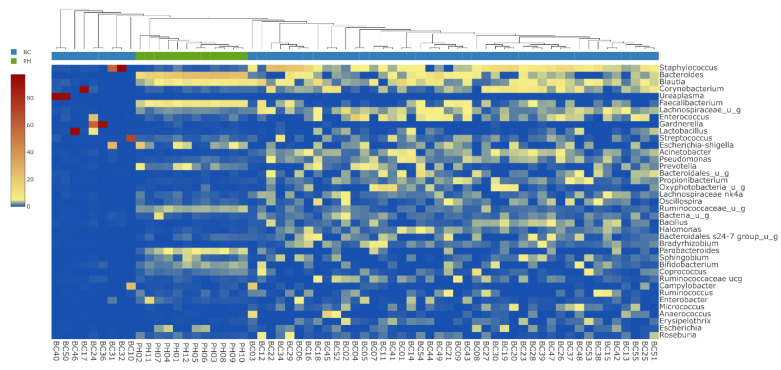
Heatmap visualization of the most abundant taxa at genus level among the BC and PH patients. The samples of the PH and BC groups are sharply separated; additionally, 9 samples of the BC group were detached in which some specific genera showed a remarkably high presence.

Each urine sample collected directly from the bladder during TUR surgery was negative by traditional routine aerobic culture. Samples of the PH and BC groups are sharply differentiated on the heatmap (Figure 2); additionally, in 9 BC samples, specific genera showed a remarkably high presence. These samples contained the following genera: BC 10: Streptococcus (69%), BC 17: Corynebacterium (93%), BC 24, 36: Gardnerella (59%, 92%), BC 31, 32: Staphylococcus (57%, 97%), BC 40, 50: Ureaplasma (95%, 93%) and BC 46: Lactobacillus (94%). Samples with these extreme outlier values—assuming an ongoing infection—were excluded from further comparative microbiome analyses and defensin expression studies.

**Figure 3:**
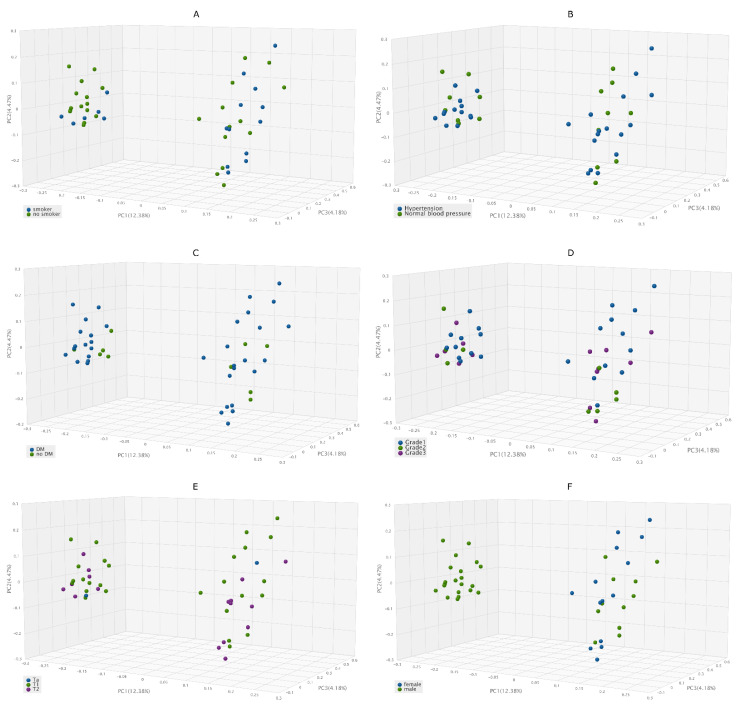
Relationship between characteristics of BC patients and microbiome beta diversity The Jaccard beta diversity graph for tumor samples shows two well-separated clusters, but the following properties alone do not show any correlation with cluster classification, (**A**) smoking habit, (**B**) hypertension, (**C**) diabetes mellitus, (**D**) grade of the cancer, (**E**) stage of the cancer. (**F**) There is a significant difference in the classification of female and male tissue samples into microbiome clusters.

After grouping the samples of the BC group by underlying patient characteristics, Figure 3A–E shows the microbiome beta diversity results with Jaccard PCoA: no significant differences were shown according to smoking habits, diagnosed hypertension or diabetes mellitus or tumor grade and stage classification. Significant differences were shown only in the case of beta diversity between samples from males and females (*p* = 0.001) (Figure 3F).

Compared to the median ΔCt value of PH samples—considered as a control group—the RQs of HBD1, HBD2 and HBD3 mRNA of PH patients and BC patients showed curves that were not normally distributed. Furthermore, 59% of samples from BC patients expressed higher amounts of HBD1. Additionally, higher HBD2 or HBD3 expression was detected in 74% or 50% of BC samples, respectively. Compared to the median values of controls, the largest differences in gene expression values in the BC group were 7-fold for HBD1, 9-fold for HBD2 and 45-fold for HBD3, respectively.

**Figure 4:**
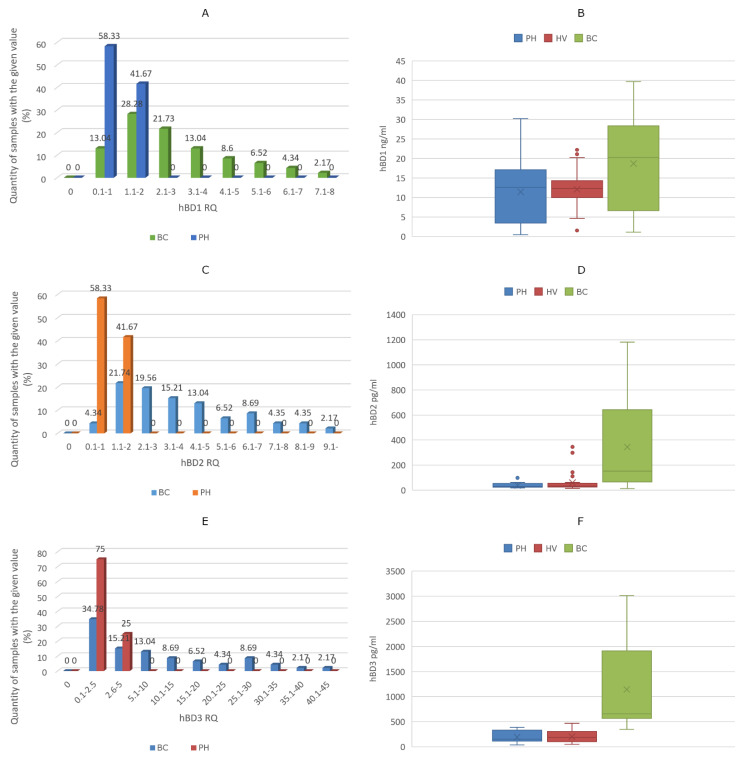
Defensin mRNA expression RQ values from tissue samples and the amounts of HBDs measured in the urine samples. (**A**) HBD1 RQ in tissue samples, (**B**) HBD1 levels in urine samples, (**C**) HBD2 RQ in tissue samples, (**D**) HBD2 levels in urine samples, (**E**) HBD3 RQ in tissue samples, (**F**) HBD3 levels in urine samples. BC: bladder cancer patients, PH: prostatic hyperplasia patients, HV: healthy volunteers.

Defensin mRNA expression RQ values and the amounts of HBDs measured in urine samples are shown in Figure 4A–E. The median amount of HBD1 was 12.59 ng/mL (IQR: 12.04), 12.33 ng/mL (IQR: 4.09) and 20.28 ng/mL (IQR: 21.44) in the PH group, HV group and BC group, respectively. Median HBD2 levels were 30.45 pg/mL (IQR: 21.93), 31.59 pg/mL (IQR: 28.88) and 151.69 pg/mL (IQR: 560.89) in the PH group, HV group and BC group, respectively. Finally, HBD3 levels were 151.96 pg/mL (IQR: 202.36), 186.44 pg/mL (IQR: 198.95) and 653.73 pg/mL (IQR: 1321) in the PH group, HV group and BC group, respectively. There were no relevant differences between the urine defensin levels between the PH and healthy control groups, with *p* values 0.65 for HBD1, 0.77 for HBD2 and 0.36 for HBD3, respectively. The HBD1 value in the BC group did not differ significantly from the PH (*p* = 0.97) or HV (*p* = 0.99) groups. In contrast, there were significant differences between the HBD2 values of the BC group, and the PH (*p* < 0.001) or HV (*p* < 0.001) groups. Additionally, HBD3 levels of BC group patients differed significantly from the PH (*p* < 0.001) or HV (*p* < 0.001) group. The increase in urinary HBD1 levels (in 32% of the samples) was not associated with the elevated amount of HBD1 mRNA expressed (58%) in excised tumor tissue; even with high mRNA expression in tumor tissue, there were low urinary HBD1 levels. Elevated tissue HBD2 mRNA expression was present in 74% of samples, and 78% of urine samples contained higher amounts of HBD2. This discrepancy was even greater for HBD3, where tumor tissue mRNA expression was increased in 50% of the samples, while the amount of HBD3 was multiplied in each urine sample.

**Figure 5:**
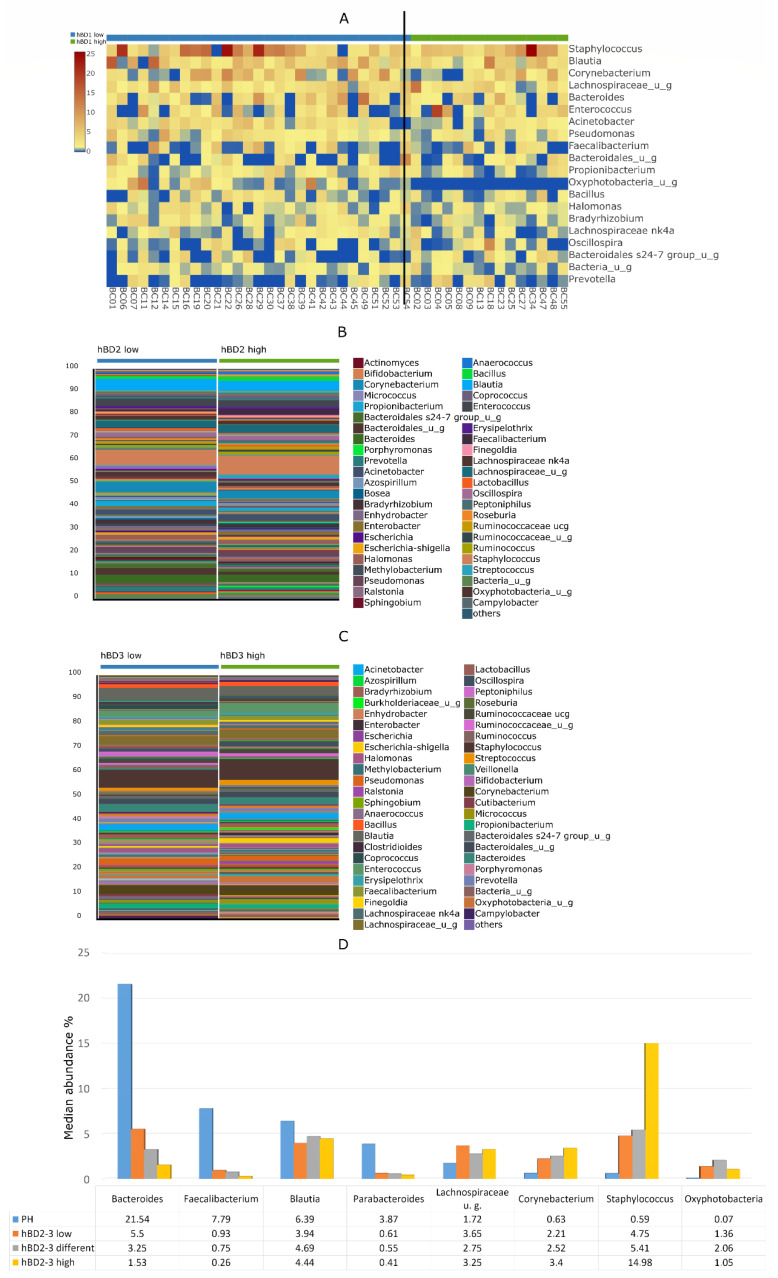
Correlation between the amount of defensins in urine and the abundance of the characteristic genera in BC tissue microbiome. (**A**) Oxyphotobacteria is the only genus whose occurrence differs significantly from the quantitative change in HBD1, Oxyphotobacteria is present only at low HBD1 levels. There is no significant difference due to the amount of (**B**) HBD2 or (**C**) HBD3 alone in the abundance of the genera in the BC tissue microbiome. (**D**) The combined increase in HBD2 and HBD3 levels reduces the abundance of non-tumor specific genera (Bacteroides, Parabacteroides, Faecalibacterium) and increases the abundance of more common in-tumor tissue genera (Staphylococcus, Corynebacterium).

The Oxyphotobacteria genus, a member of the Cyanobacteria phylum, presents only in the tissue microbiome of patients with low urinary HBD1 values. With the exception of Oxyphotobacteria, no other genus abundance showed an association with either HBD1, HBD2 or HBD3 levels separately (Figure 5A–C). In addition to the combined quantitative change in HBD2 and HBD3, there was a clear trend in the change in abundance of Bacteroides, Parabacteroides, Faecalibacterium, Corynebacterium and Staphylococcus genera (Figure 5D). Compared to the comparator PH group, the tumor-specific low levels of Bacteroides, Parabacteroides and Faecalibacterium gradually decreased with the combined increase in HBD2 and HBD3. Higher Corynebacterium and Staphylococcus abundance in BC samples increased in parallel with an increase in HBD2 and HBD3 levels. No such association was found regarding the abundance of Blautia, Lachnospiraceae and Oxyphotobacteria genera.

## 4. Discussion

BCG (*Mycobacterium bovis*, Bacillus Calmette–Guérin) has been used as one of the most successful anti-tumor immunotherapies since 1976 [22]. This specific strain of the *Mycobacterium* genus not only works against BC by activating the immune system, but also directly reacts to the tumor cells, causing apoptosis [23], necrosis [24] or oxidative stress [25]. One possible reason for the lower incidence of BC in women may be the significantly higher incidence of *Actinomycetes* (including the *Mycobacterium* genus) in the female urinary microbiome [26]. Only a fraction of the microbes present in the bladder were previously detectable by conventional urine culture methods, however, advances in molecular techniques have made it possible to quantify and qualitatively assess a large number of bacterial DNAs from urine or bladder tissue [7,8,10,27,28,29,30]. An individual’s pre-existing bladder microbiome may have a role not only in the development of BC [30], or in protection against cancer formation [26], but also in their response to immunotherapy [7]. The surrounding microbiome also plays a role in treatment failure with BCG intravesical instillation or with anti-PD-1/PD-L1 immune checkpoint inhibitors. Thus, the higher amount of HBD2 induced by the bacteria present in the microbiome impairs the efficacy of BCG therapy [16,17,31]. Chen et al. demonstrated a significant relationship between the amount of *Leptotrichia*, *Roseomonas*, *Propionibacterium*, *Prevotella* and *Massilia* genera in the microbiome of BC tissue and the PD-L1 expression of tumor cells [18]. Based on the experience of retrospective studies, it was found that the effectiveness of various checkpoint inhibitors was significantly reduced by different antibiotics when used in parallel; this effect may also be explained by distinct changes in microbial composition that are detrimental to therapeutic effectiveness of these anticancer medications [32,33].

Although no bacteria were cultured from any of the urine samples, nine tissue samples contained *Streptococcus*, *Corynebacterium*, *Gardnerella*, *Staphylococcus*, *Ureaplasma* or *Lactobacillus* in remarkably high rates. There may be several reasons for the discrepancy, such as the difference between the tissue and the urine microbiome, or more likely, the unsuitability of conventional culture conditions. As an existing urinary tract infection—that could result in a shift in defensin values—could not be ruled out in these samples, defensin expression and ELISA assays were not performed on these samples. 

As a result of our previous research, we have demonstrated that the microbiome composition of tumor tissue and the surrounding urine shows characteristic differences [19]. In our previous study, we hypothesized a role in tumor formation or progression, based on the much higher incidence of *Akkermansia*, *Bacteroides*, *Clostridium sensu stricto*, *Enterobacter* and *Klebsiella* in tumor tissue compared to urine. We now interpret our results that the *Bacteroides* genus is associated with the mucosa rather than free urine, as in our current study we examined whether the composition of mucosa-associated bacteria differs in tumor (BC) and non-tumor (PH) tissue samples. We have shown pronounced differences in both alpha and beta diversity between the two sample types. In BC patients, the abundance of *Staphylococcus*, *Corynebacterium* and *Oxyphotobacteria* genera was noted, while in PH patients, *Faecalibacterium* and *Bacteroides* abundances were significantly higher. Reports show that *Faecalibacterium*—which has shown low abundance levels in tumor tissue—has a protective effect against colon, prostate or even breast cancer, if present in high abundance in feces [34,35,36]. 

In contrast to Ma et al., who found a significant difference in the urinary microbiome beta diversity of BC patients between smokers and non-smokers [37], there was no difference examined in our tissue samples. We also found no difference in tissue microbiome beta diversity based on associations with diabetes, hypertension or tumor grade and stage. Pederzoli et al. investigated gender-related differences in the urine of BC and healthy patients, and in tumor and healthy tissue samples [38]. Confirming our previous study [19], only around 80% of the bacterial taxa were found in the urine, compared to what was detected in the tissue. In our previous study [19] with a small number of samples, a significant difference was observed in the tissue samples of five males and five females in both α and ß diversity of the microbiome. In our present study, the microbiome ß diversity of the 32 male and 14 female tumor samples also showed significant differences; on the other hand, Pederzolli et al. examined 21 male and 8 female tumor samples and found no significant differences in their microbial compositions.

To the best of our knowledge, this is the first study that looks for a correlation between defensin-levels and the composition of the tissue microbiome in patients with BC. Nienhouse et al. found that patients with a lower amount of HBD1 have a higher risk of postoperative urinary infection, especially for infections caused by Gram-negative bacteria, while no similar correlation was found in the case of HBD2 [39]. In the HV group, only urinary HBD levels were determined, but as there were no significant differences between the PH and HV groups, defensin expression values of the PH group were used as a negative control to characterize the expression of tumor tissues. HBD1 is constitutively expressed in urogenital tract epithelia; however, its altered expression has been described in multiple human cancers [15]. Although 58% of our tumor tissue samples showed increased HBD1 expression, HBD1 content of patients’ urine was not significantly elevated. It is hypothesized that these patients genetically produce less HBD1 in the healthy area of the urinary tract. As HBD1 is a natural tumor inhibitor [14,15], these patients may be genetically more prone to developing tumors. Moreover, the amount of *Oxyphotobacteria*—which is a confirmed tumor-causing *Cyanobacterium* genus [40]—was significantly higher in the BC group, and its presence was associated with low HBD1 levels.

BC cells also showed higher HBD2 and HBD3 expression-levels, but the urinary levels of defensins were also substantially higher, thus, the expression of inducible defensins is also likely to be elevated in other urothelial cells. Microorganisms and inducible defensins mutually affect each other’s presence in the bladder; the resulting balance may also be affected by a number of external factors. Examination of HBD2 or HBD3 levels individually showed no association with microbial composition, but the combined effect of HBD2 and HBD3 [41] shows an increasingly pronounced difference in the abundance of the genera characteristic for the PH and BC groups; i.e., the higher the HBD2 and 3 levels, the greater the differences in the microbiome composition between the two groups. HBD3 is also bactericidal against some Gram-positive species, in addition to its ability to kill Gram-negative bacteria, which is also characteristic of HBD1–2 [42]. Our present study also highlights the phenomenon that, with high HBD2–3 levels, Gram-negative bacteria appear with a lower abundance compared to the healthy state. This situation may be considered as a “snapshot”, possibly showing a new state of equilibrium characteristic of the tumor, but possibly an episode in a vicious cycle of ever-deteriorating conditions in microbiome composition.

## 5. Conclusions

Among other factors, defensins and microorganisms also affect the development, progression and treatment options for bladder cancer. The amount of defensins measured in the urine depends not only on the expression levels in the tumor, but also in other areas of the urinary tract. Based on our findings, there is a significant difference between tumor and non-tumor microbiome composition. In addition, the urine cancer patients showed decreased levels of antitumor HBD1, and increased levels of HBD2 and HBD3, which also corresponded to the characteristic microbial composition of the tumors. To enhance the success of immunotherapies and to develop adjuvant antitumor therapies, it is important to gain insight into the interactions between defensins and the tumor-associated microbiome.

## Data Availability

The datasets generated and analysed during the current study are available in the SRA repository: SRA/ PRJNA 809202/www.ncbi.nlm.nih.gov (accessed on 22 February 2022).

## Supplementary Materials

The following supporting information can be downloaded at: https://www.mdpi.com/article/10.3390/biomedicines10071758/s1, Table S1: The characteristics of the study participants.
