# Supplementary material for: Bladder Tissue Microbiome Composition in Patients of Bladder Cancer or Benign Prostatic Hyperplasia and Related Human Beta Defensin Levels
Source: Biomedicines. 2022 Jul 21;10(7):1758. doi: 10.3390/biomedicines10071758 (PMC9313236; doi:10.3390/biomedicines10071758)
Supplement: Supplementary file 1 [file biomedicines-10-01758-s001.zip › biomedicines-1789471-supplementary.pdf]

Supplementary material S1

Bladder tissue microbiome composition in patients of bladder cancer or benign prostatic hyperplasia and related human beta defensin levels

Mansour Bassel[1], Ádám Monyók[1], Márió Gajdács[2], Balázs Stercz[3], Nóra Makra[3], Kinga Pénzes[3], István Vadnay[4], Dóra Szabó[3], Eszter Ostorházi[3,5,*]

Table S1. The characteristics of the study participants.

	**Bladder Cancer Group**	**Prostatic Hypertrophy**	**Healthy Volunteers**
**Number of participants**	55 (46 in defensin tests)	12	34
**Median age of participants**	68.5 (IQR: 14)	71.5 (IQR:19)	51 (IQR:42)
**Male/Female**	32/14	12	17/17
**Diabetes mellitus/ No DM**	36/10	8/4	1/33
**Hypertension/ Normal blood pressure**	29/17	9/3	4/30
**Smoker/No smoker**	28/18	7/5	12/24
**Stage:**	Ta:2, T1:27, T2:17,	NA	NA
**Grade:**	G1:23 ,G2:9, G3:14	NA	NA
